# Correction to: Reactivation of TAp73 tumor suppressor by protoporphyrin IX, a metabolite of aminolevulinic acid, induces apoptosis in *TP*53-deficient cancer cells

**DOI:** 10.1186/s13008-019-0052-x

**Published:** 2019-08-29

**Authors:** Alicja Sznarkowska, Anna Kostecka, Anna Kawiak, Pilar Acedo, Mattia Lion, Alberto Inga, Joanna Zawacka‑Pankau

**Affiliations:** 1 Department of Biotechnology, Intercollegiate Faculty of Biotechnology, University of Gdansk and Medical University of Gdansk, Abrahama 58, 80-307 Gdansk, Poland; 2 Department of Microbiology, Tumor and Cell Biology, Karolinska Institutet, Biomedicum, Solnavägen 9, 171 65 Stockholm, Sweden; 3 Centre for Integrative Biology, CIBIO, University of Trento, via Sommarive 9, 38123 Trento, Italy; 4 Present Address: Department of Molecular Biology, Massachusetts General Hospital, Harvard Medical School, Boston, MA 02114 USA

## Correction to: Cell Div (2018) 13:10 10.1186/s13008-018-0043-3

The authors note a correction to the article [1]. Figure 1d of the original article has an error. The pcDNA3/Ctrl and the pcDNA3-TAp73a/DMSO wells are duplicated. This article presents the corrected version of Fig. 1.

**Fig. 1:**
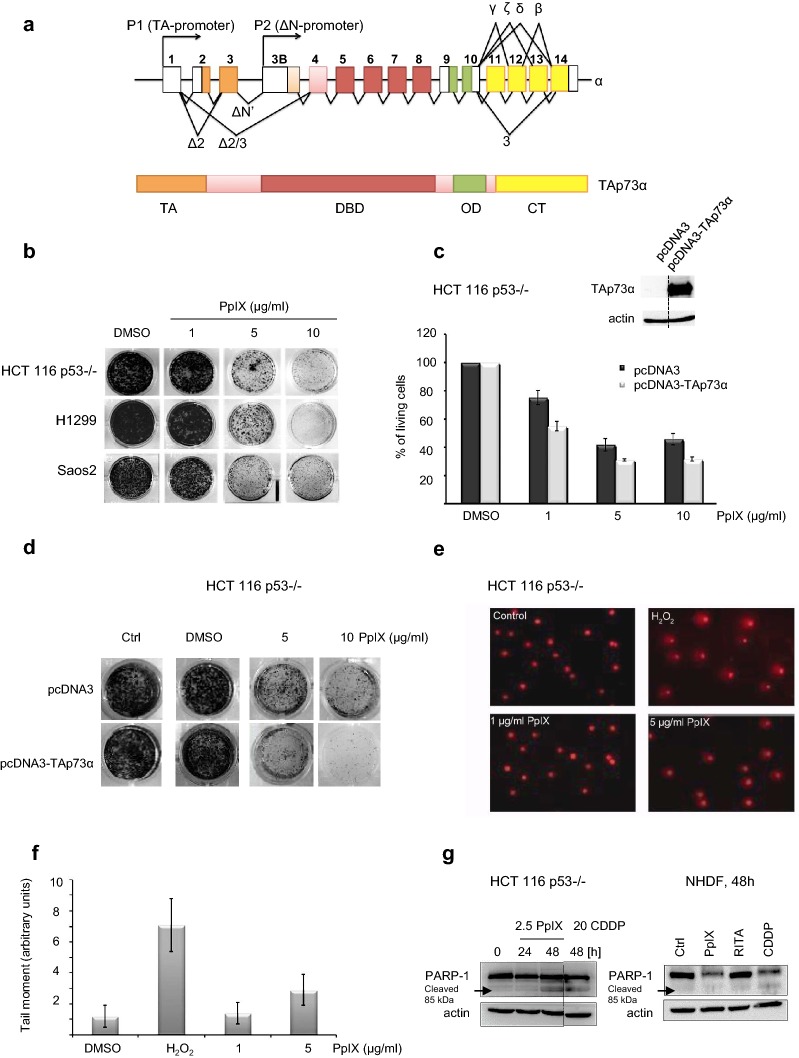
PpIX inhibits proliferation of cancer cells lacking p53. **a** Schematic representation of the splice variants in TP73 gene (upper panel) and domain structure of p73 (lower panel). *TA* transactivation domain, *DBD* DNA binding domain, *OD* oligomerization domain, *CTC* terminus. **b** PpIX induces dose-dependent growth inhibition in a long-term proliferation assay. **c** Ectopic expression of TAp73α sensitizes cells to PpIX after 24 h as demonstrated by WST-1 proliferation assay. Inserted blot represents the level of expression of TAp73α. Please note that the blot has been cropped. Dotted line represents where the blot has been cut. The uncropped full length version is presented in Additional file 3: Figure S3a. **d** TAp73α overexpression sensitizes H1299 to PpIX-induced inhibition of proliferation. **e**, **f** PpIX does not induce DNA damage in cancer cells at the effective therapeutic concentrations. **g** 2.5 μg/ml PpIX induces PARP-1 cleavage in HCT 116 p53−/− but not in non-transformed human diploid fibroblasts. Dotted line represents where the blot has been cut. The uncropped blot is presented in Additional file 3: Figure S3b
