# Supplementary figures and images for: Reactivation of TAp73 tumor suppressor by protoporphyrin IX, a metabolite of aminolevulinic acid, induces apoptosis in TP53-deficient cancer cells
Source: Cell Div. 2018 Dec 26;13:10. doi: 10.1186/s13008-018-0043-3 (PMC6306007; doi:10.1186/s13008-018-0043-3)

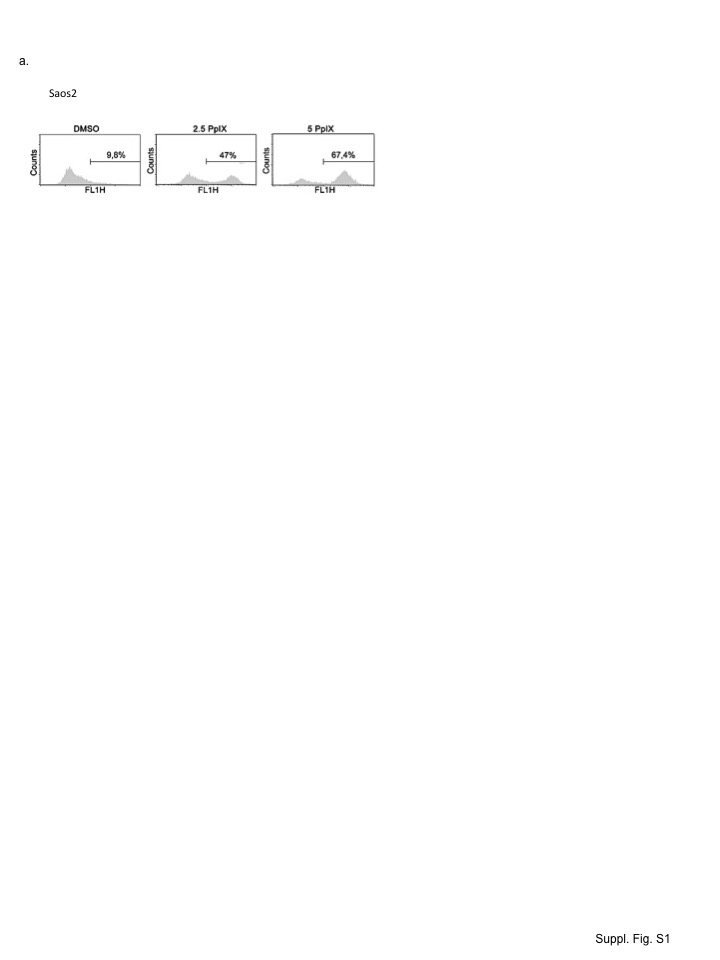

Supplement: Supplementary file 1 — Additional file 1: Figure S1. (a) PpIX induces caspases as shown as the increase in the fluorescent signal in p53-null Saos2 cells. [file 13008_2018_43_MOESM1_ESM.jpg]

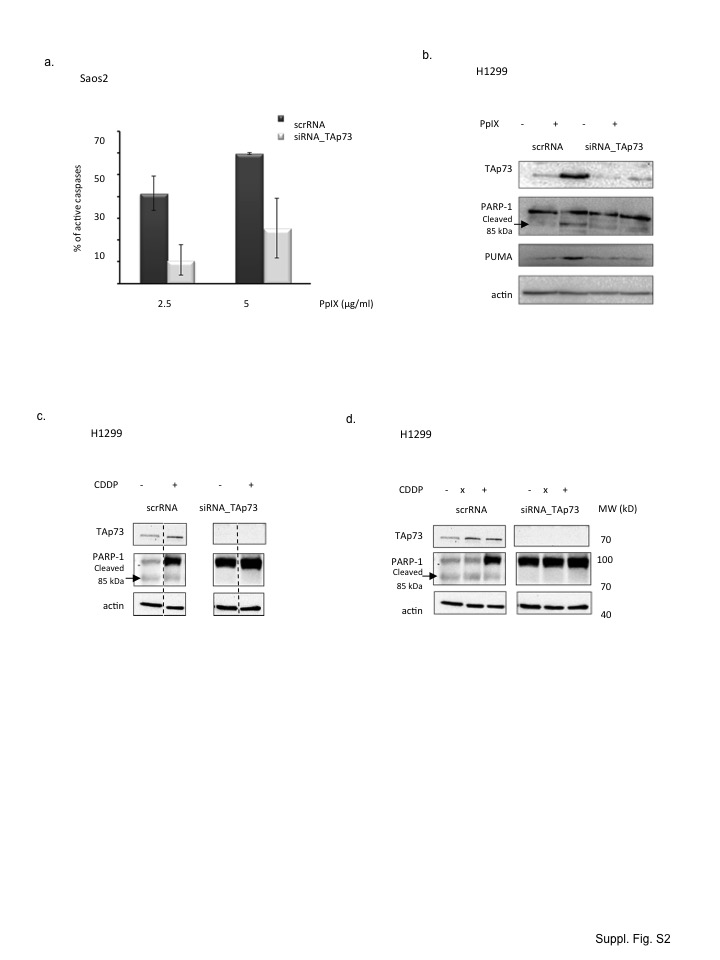

Supplement: Supplementary file 2 — Additional file 2: Figure S2. (a) TAp73 depletion protects from PpIX-induced caspase activation in p53-null Saos2 cells. (b) TAp73 knockdown ablates PpIX-induced accumulation of PUMA and PARP-1 cleavage in H1299 cells. (c) Silencing of TAp73 partially inhibits PARP-1 cleavage in H1299 after CDDP treatment. Dotted line indicates sites where membrane was cut. The uncut blots are shown in (d). [file 13008_2018_43_MOESM2_ESM.jpg]

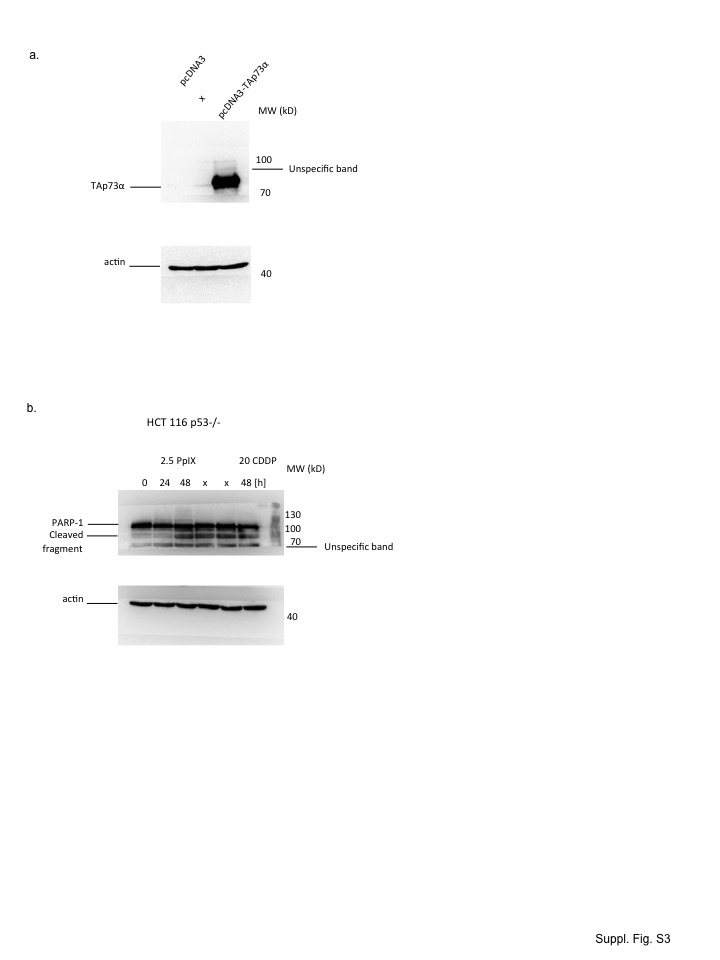

Supplement: Supplementary file 3 — Additional file 3: Figure S3. Uncropped versions of the blots presented in Fig. 1b and f, respectively. X-sample or compound not included in this study. [file 13008_2018_43_MOESM3_ESM.jpg]

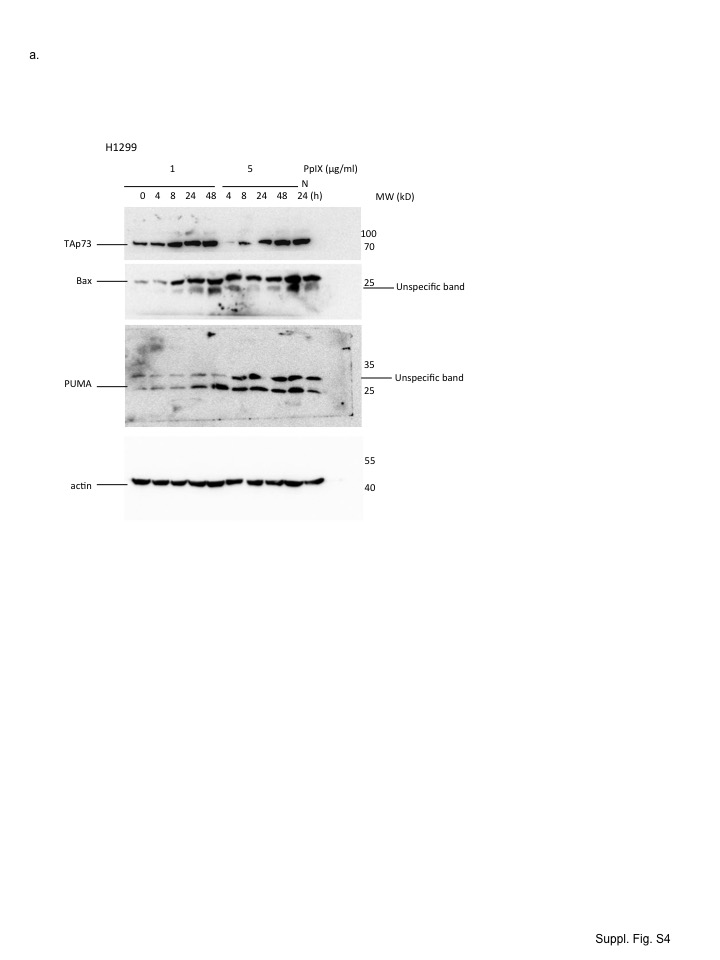

Supplement: Supplementary file 4 — Additional file 4: Figure S4. Uncropped versions of the blots presented in Fig. 2d. [file 13008_2018_43_MOESM4_ESM.jpg]

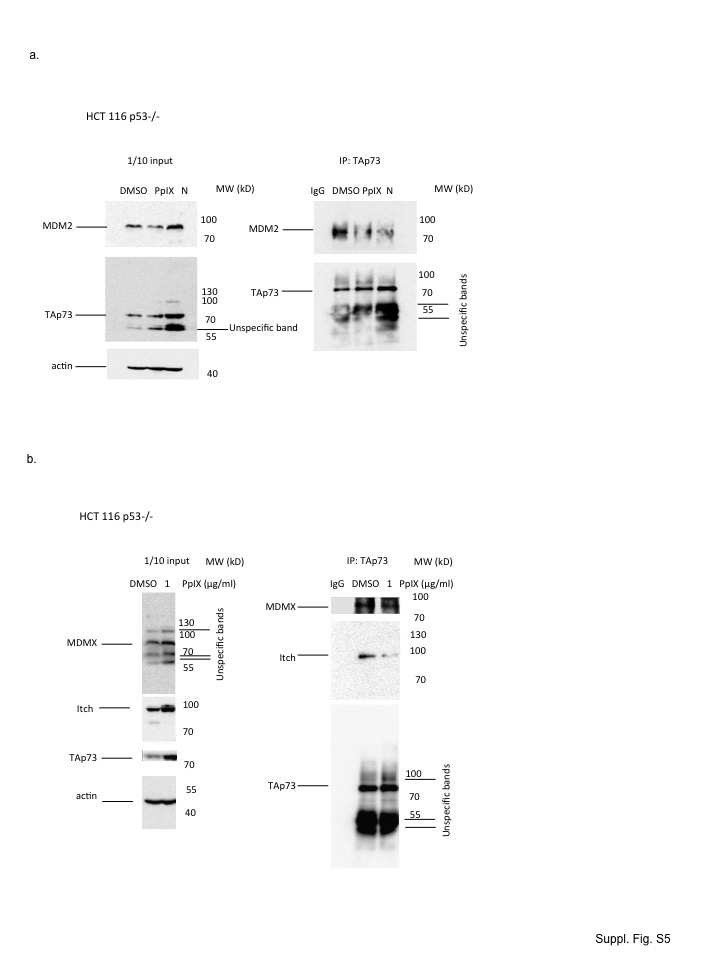

Supplement: Supplementary file 5 — Additional file 5: Figure S5. Uncropped versions of the blots presented in Fig. 4b, c. [file 13008_2018_43_MOESM5_ESM.jpg]

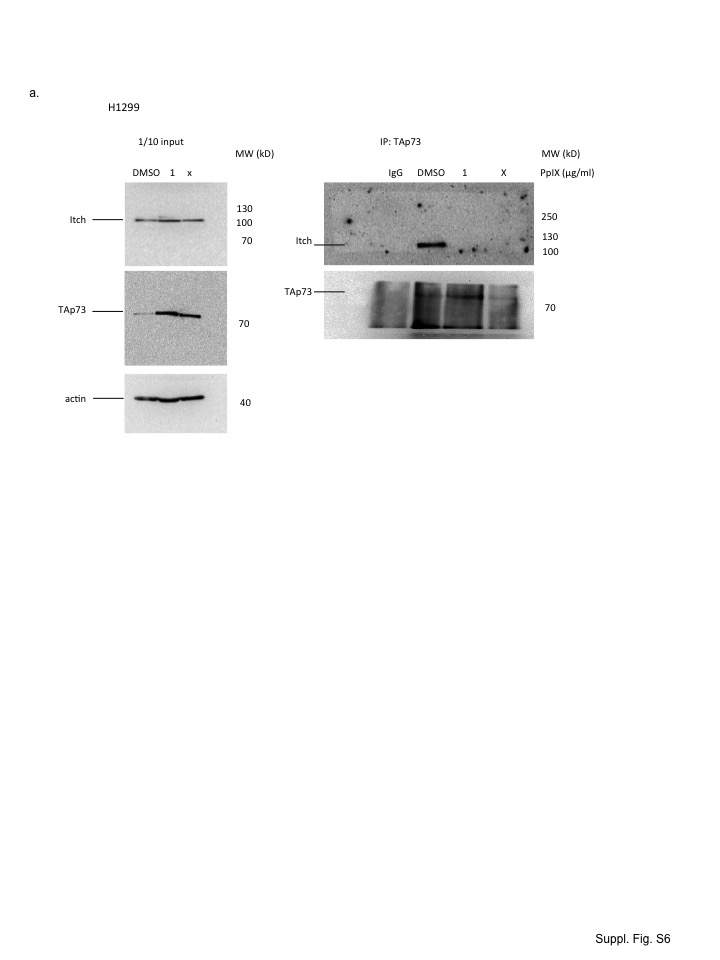

Supplement: Supplementary file 6 — Additional file 6: Figure S6. Uncropped versions of the blots presented in Fig. 4e. [file 13008_2018_43_MOESM6_ESM.jpg]
